# Association of the Hemoglobin–Albumin–Lymphocyte–Platelet (HALP) Score with 3-Month Outcomes After Lumbar Medial Branch Radiofrequency Ablation: A Retrospective Cohort Study

**DOI:** 10.3390/diagnostics15212758

**Published:** 2025-10-31

**Authors:** Çile Aktan, Gözde Çelik, Cemil Aktan

**Affiliations:** 1 Department of Pain Medicine, Antalya Training and Research Hospital, 07100 Antalya, Türkiye; drcilezengin@hotmail.com; 2 Department of Pain Medicine, Malatya Training and Research Hospital, 44000 Malatya, Türkiye; gozdecelik555@gmail.com; 3 Department of Orthopaedics and Traumatology, Antalya Training and Research Hospital, 07100 Antalya, Türkiye

**Keywords:** hemoglobin–albumin–lymphocyte–platelet (HALP), radiofrequency ablation, medial branch, facet joint, chronic low back pain, visual analog scale, Oswestry Disability Index, prognostic biomarker

## Abstract

**Background:** The hemoglobin–albumin–lymphocyte–platelet (HALP) score integrates the immunonutritional and inflammatory status. We evaluated whether baseline HALP predicts the 3-month response after lumbar medial branch radiofrequency ablation (RFA), defined as a Visual Analogue Scale (VAS) reduction of ≥50% and an Oswestry Disability Index (ODI) reduction of ≥40%, and identified a Youden-optimal cut-off. The discrimination and calibration of multivariable models were also assessed. **Methods:** This single-center retrospective cohort (*N* = 120) included rigorously selected patients (≥50% pain relief after two comparative medial branch blocks) undergoing standardized RFA. Multivariable logistic regression was adjusted for age, sex, Body Mass Index (BMI), smoking status, paraspinal tenderness, and baseline scores. We quantified the Area Under the Receiver Operating Characteristic Curve (AUC), Hosmer–Lemeshow (HL) goodness-of-fit, Brier score, and calibration slope; optimism was corrected using a 500-bootstrap method. **Results:** Responses occurred in 64.2% (VAS) and 65.8% (ODI) of participants. HALP independently predicted ODI (OR = 1.06, 95% CI 1.02–1.09; *p* < 0.001) and VAS (OR = 1.05, 95% CI 1.02–1.08; *p* = 0.001). As a single predictor, HALP showed fair discrimination (AUC 0.717 [VAS], 0.731 [ODI]). The Youden cut-off of 39.8 yielded high sensitivity (~0.87) with modest specificity (~0.58–0.61). Multivariable AUCs were 0.744 (VAS) and 0.774 (ODI), optimism-corrected to 0.680 and 0.720; calibration was acceptable (HL *p* > 0.05; slopes ≈ 0.74–0.78; Brier 0.188/0.179). **Conclusions:** HALP is a simple, low-cost adjunct that independently predicts short-term pain and functional outcomes after lumbar medial branch RFA. Incorporation into post-block triage may refine selection, especially for functional improvement, pending prospective external validation and recalibration of the cut-off.

## 1. Introduction

Chronic low back pain is one of the leading causes of disability and loss of workforce worldwide. Its prevalence increases with age and imposes a substantial economic burden on healthcare systems. Facet joint pathologies are considered responsible for approximately one-third of chronic spinal pain cases [1]. Degenerative changes, repetitive microtrauma, and age-related structural alterations represent the primary underlying mechanisms of facet joint pain. In these patients, conservative treatments are often insufficient, and interventional procedures become the preferred options.

Among the most frequently applied interventional treatments for facet-mediated pain is medial branch radiofrequency ablation (RFA) [2]. Although this procedure has demonstrated high success rates in appropriately selected patients, it remains challenging in clinical practice to predict in advance which patients will benefit from treatment. Diagnostic medial branch blocks are used to identify potential responders, yet they do not reliably guarantee long-term treatment success. Therefore, the lack of reliable predictive markers continues to represent a significant clinical gap in the management of facet joint pain.

From a pathophysiological perspective, chronic low back pain, primarily when arising from degenerative facet joints, is increasingly recognized as involving persistent low-grade inflammation. Cytokines such as IL-1β, IL-6, and TNF-α sensitize nociceptors and amplify central sensitization [3]. Consequently, systemic inflammatory and immunonutritional markers have attracted growing interest as potential predictors of treatment response in interventional pain management.

Several hematological indices have been explored in this context. The neutrophil-to-lymphocyte ratio (NLR), platelet-to-lymphocyte ratio (PLR), and systemic immune-inflammation index (SII) have each been associated with outcomes in musculoskeletal and degenerative disorders [4]. For example, NLR has been reported as an early predictor of surgical site infection after spine surgery [5] and as a prognostic marker in orthopedic and musculoskeletal conditions such as impaired gait performance and sarcopenia [6]. However, these markers mainly capture inflammatory cell-line dynamics while overlooking nutritional and oxygen-carrying capacity, which are equally relevant to pain processing and tissue recovery [7].

By contrast, the hemoglobin–albumin–lymphocyte–platelet (HALP) score integrates hemoglobin, albumin, lymphocytes, and platelets into a composite index, thus reflecting not only systemic inflammation but also nutritional and immune reserve. The HALP index combines hemoglobin, albumin, and lymphocyte counts, reflecting oxygen-carrying, nutritional, and immune reserves, divided by platelet count, which mirrors proinflammatory and prothrombotic activity. This multiplicative formulation expresses the balance between restorative and inflammatory processes that influence pain modulation and tissue recovery. Recent evidence has shown that HALP demonstrates strong prognostic performance in diverse conditions beyond oncology, such as predicting mortality in acute pancreatitis [8,9,10]. This broader scope may provide additional predictive value in procedures such as medial branch RFA, where both inflammatory control and tissue healing influence clinical outcomes.

## 2. Materials and Methods

### 2.1. Study Design and Setting

This retrospective observational study was conducted at a pain clinic between June 2023 and June 2025. The study protocol was approved by the Antalya Training and Research Hospital Clinical Research Ethics Committee (Approval No. 13/2, dated 31 July 2025) and conducted in accordance with the principles outlined in the Declaration of Helsinki.

### 2.2. Participants

**Figure 1:**
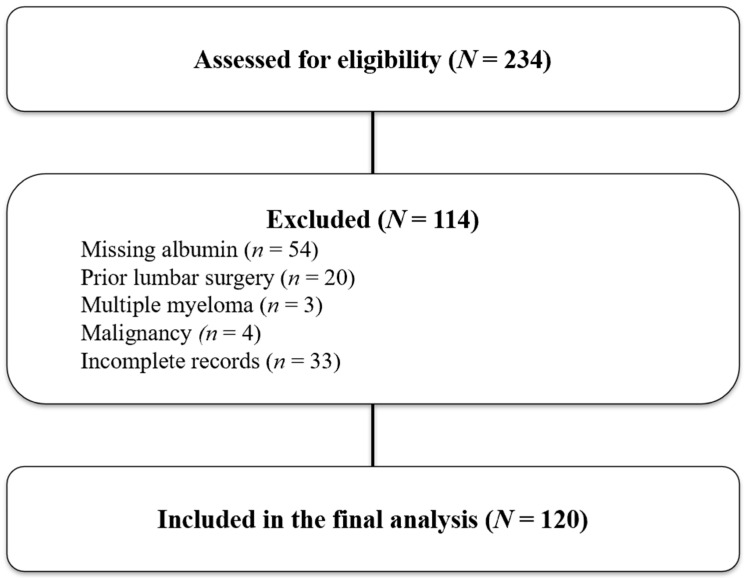
Study flow diagram showing patient selection for the final cohort. A total of 234 patients were screened for eligibility. Of these, 114 were excluded: 54 due to missing albumin values, 20 with prior lumbar surgeries, 3 with multiple myeloma, 4 with malignancy, and 33 with incomplete records. Finally, 120 patients met the criteria and were included in the final analysis.

The study included patients aged 18 years or older with chronic low back pain attributed to degenerative facet joint pathology. Facet-mediated pain was confirmed by two diagnostic medial branch blocks (MBBs), each producing ≥50% temporary pain relief. Eligible participants had persistent symptoms for at least three months despite conservative treatments such as pharmacotherapy and physiotherapy, had complete blood test results including hemoglobin, albumin, lymphocyte, and platelet counts within one week before the RFA procedure, and had both Visual Analog Scale (VAS) and Oswestry Disability Index (ODI) scores recorded at baseline and at 3-month post-treatment. Patients were excluded if they had undergone prior lumbar spine surgery at the target levels, had active infection, systemic inflammatory or autoimmune diseases, malignancy, hematologic disorders, or advanced hepatic or renal failure. Those with incomplete medical records or missing follow-up data were also excluded. Of the 234 patients initially screened, 114 were excluded due to missing pre-procedure albumin tests (*n* = 54), prior lumbar spinal surgery (*n* = 20), multiple myeloma (*n* = 3), a history of malignancy (*n* = 4), or incomplete clinical records (*n* = 33). A total of 120 patients were therefore included in the final analysis (Figure 1).

### 2.3. Procedures

**Figure 2:**
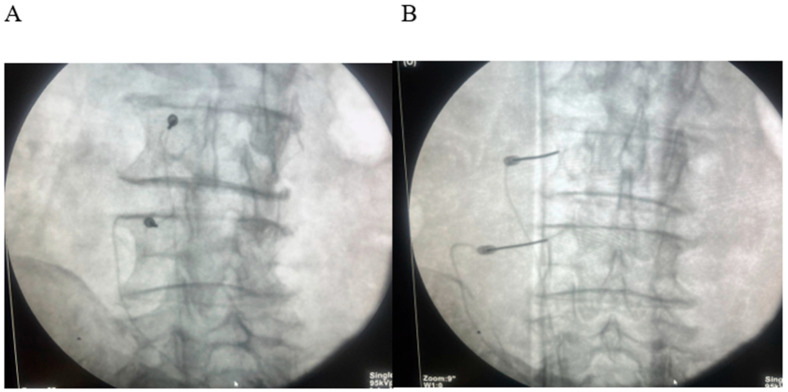
(**A**) Oblique fluoroscopic “Scotty-dog” view demonstrating tunnel-view alignment of the radiofrequency (RF) cannula along the target trajectory of the lumbar medial branch. (**B**) Anteroposterior (AP) fluoroscopic view confirming accurate placement of the RF cannula tip at the junction of the superior articular process and transverse process.

All patients underwent fluoroscopy-guided lumbar medial branch RFA under aseptic conditions. After local anesthesia, a 22-gauge RF cannula with a 10 mm active tip (Boston Scientific, Marlborough, MA, USA) was advanced to the target medial branch nerve at the junction of the transverse process and superior articular process (Figure 2).

Sensory and motor stimulation were performed to confirm correct positioning. Continuous RFA was applied at 80 °C for 90 s at each target.

Typically, two to three lumbar levels were treated per patient, most frequently involving the L4–L5 and L5–S1 levels, based on clinical findings, imaging results, and diagnostic block outcomes.

### 2.4. HALP Score Calculation

Baseline HALP scores were calculated as:

HALP = Hemoglobin (g/L) × Albumin (g/L) × Lymphocyte count (10^9^/L)/Platelet count (10^9^/L)

Laboratory measurements were performed using standardized automated analyzers in the hospital’s central laboratory. Hemoglobin values reported in g/dL were converted to g/L before HALP computation.

### 2.5. Outcome Measures

Primary outcomes were changes in pain intensity (VAS) and disability (ODI) at 3 months post-RFA compared to baseline.

A good response was defined as VAS ≥ 50% and ODI ≥ 40% reduction from baseline; ODI values are presented as raw points (0–50), with the reduction, and thus responder status computed from the baseline raw score.

### 2.6. Statistical Analyses

All analyses were performed in IBM SPSS Statistics v27 (IBM Corp., Armonk, NY, USA). Continuous variables are summarized as mean ± SD and compared using the Mann–Whitney U test; categorical variables as counts (%) and compared using Pearson’s χ^2^ test (Fisher’s exact test when appropriate). Odds ratios with 95% confidence intervals were obtained from Crosstabs (Risk). Associations between baseline HALP and outcome changes were assessed using Spearman’s rank correlation (ρ); two-sided *p*-values were reported. Receiver operating characteristic (ROC) analyses were performed for HALP and for model-predicted probabilities; the Youden index was used to determine the optimal threshold. Model calibration was assessed using the Hosmer–Lemeshow test (g = 10), the Brier score (mean squared error of predicted probabilities), and the calibration slope estimated by logistic regression of the outcome on the logit of the expected probability. Internal validation employed bootstrap resampling (*n* = 500) to obtain optimism-corrected AUC and calibration slope. Because the dependent variable (treatment responder = yes/no) was binary, logistic regression was used, which estimates associations as odds ratios (ORs) rather than risk ratios (RRs).

## 3. Results

A total of 120 patients were analyzed according to VAS and ODI response criteria. Based on the VAS criterion (≥50% reduction), 77 patients (64.2%) were classified as responders and 43 (35.8%) as non-responders. The mean age did not differ significantly between responders and non-responders (68.81 ± 5.90 vs. 68.77 ± 7.20 years, *p* = 0.773). The female sex distribution was similar in the two groups (54.5% [42/77] vs. 60.5% [26/43], *p* = 0.663). Baseline VAS scores were slightly higher in responders (8.60 ± 0.91 vs. 8.30 ± 0.86), but this difference did not reach statistical significance (*p* = 0.130). Likewise, baseline ODI scores (22.14 ± 4.99 vs. 20.95 ± 3.55, *p* = 0.510) and BMI (30.37 ± 2.62 vs. 30.72 ± 3.15, *p* = 0.368) did not differ between groups. Paraspinal tenderness was observed in 85.7% (66/77) of responders compared with 72.1% (31/43) of non-responders (*p* = 0.115). Smoking rates were also comparable (49.4% [38/77] vs. 41.9% [18/43], *p* = 0.550). In contrast, the mean HALP score was significantly higher among responders compared with non-responders (54.61 ± 14.96 vs. 43.35 ± 14.68, *p* < 0.001) (Table 1).

When classified according to the ODI criterion (≥40% improvement), 79 patients (65.8%) were identified as responders and 41 (34.2%) as non-responders. Similarly to the VAS-based analysis, mean age (68.73 ± 5.84 vs. 68.90 ± 7.34 years, *p* = 0.898), sex distribution (55.7% vs. 58.5% female, *p* = 0.918), baseline ODI (22.06 ± 4.95 vs. 21.05 ± 3.60, *p* = 0.660), and BMI (30.34 ± 2.64 vs. 30.78 ± 3.15, *p* = 0.318) showed no significant group differences. Baseline VAS was slightly higher in responders (8.61 ± 0.90 vs. 8.27 ± 0.87) and demonstrated a borderline statistical association (*p* = 0.067). Paraspinal tenderness was more frequent among responders (86.1% [68/79] vs. 70.7% [29/41]), showing a non-significant trend (*p* = 0.075). Smoking prevalence did not differ significantly (49.4% [39/79] vs. 41.5% [17/41], *p* = 0.529). Consistent with the VAS-based analysis, HALP scores were significantly higher in responders compared with non-responders (54.65 ± 14.83 vs. 42.72 ± 14.62, *p* < 0.001) (Table 2).

Correlation analysis revealed a positive association between baseline HALP scores and both pain reduction and functional improvement. HALP was significantly correlated with ΔVAS (ρ = 0.238, *p* = 0.009) and ΔODI (ρ = 0.277, *p* = 0.002). Furthermore, ΔVAS and ΔODI were strongly associated with each other (ρ = 0.808, *p* < 0.001), indicating that pain relief and functional improvement progressed in parallel.

### 3.1. Logistic Regression Analyses

According to the ODI response criterion, multivariable logistic regression analysis demonstrated that the HALP score was significantly associated with treatment response (OR = 1.06, 95% CI: 1.02–1.09, *p* <0.001). Age (OR = 1.00, 95% CI: 0.94–1.07, *p* = 0.952), sex (OR = 0.97, 95% CI: 0.42–2.26, *p* = 0.948), BMI (OR = 0.98, 95% CI: 0.85–1.13, *p* = 0.779), smoking (OR = 1.22, 95% CI: 0.53–2.82, *p* = 0.645), and paraspinal tenderness (OR = 2.13, 95% CI: 0.78–5.88, *p* = 0.142) were not significantly associated with ODI response (Table 3).

When patients were analyzed according to the VAS response criterion, the HALP score again emerged as a significant independent predictor (OR = 1.05, 95% CI: 1.02–1.08, *p* = 0.001). In contrast, age (OR = 1.01, 95% CI: 0.95–1.08, *p* = 0.793), sex (OR = 1.14, 95% CI: 0.50–2.61, *p* = 0.761), BMI (OR = 0.99, 95% CI: 0.86–1.14, *p* = 0.874), and smoking (OR = 1.21, 95% CI: 0.53–2.75, *p* = 0.647) were not significantly associated with VAS response. Paraspinal tenderness showed a trend toward higher odds of VAS response (OR = 1.96, 95% CI: 0.72–5.34), but did not reach statistical significance (*p* = 0.190) (Table 4).

Overall, both models consistently indicated that a higher baseline HALP score independently predicted favorable treatment outcomes, whereas demographic and clinical covariates had no significant effect.

Stratified analysis by the Youden-optimal HALP cut-off (39.8) further supported these findings. Baseline characteristics by HALP group are provided in Supplementary Table S1; notably, baseline ODI was modestly higher in patients with HALP < 39.8 compared with those with HALP ≥ 39.8 (23.03 ± 4.53 vs. 21.15 ± 4.47; Mann–Whitney U, *p* = 0.026). Considering HALP ≥ 39.8 as favorable, responder rates were higher in the high-HALP group for both endpoints (VAS ≥ 50% and ODI ≥ 40%); exact counts, *p*-values, and crude odds ratios with 95% CIs are provided in Supplementary Table S2.

### 3.2. ROC/Discrimination Analyses

**Figure 3:**
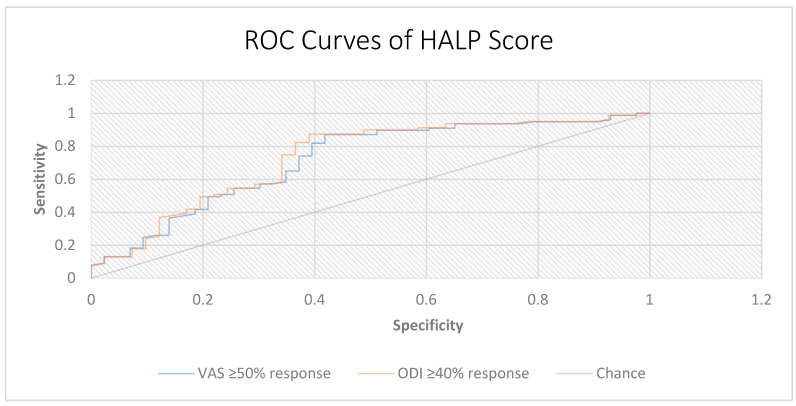
Receiver operating characteristic (ROC) curves of the HALP score for predicting treatment response. ROC analysis of HALP score in predicting clinical response according to pain and disability outcomes. Treatment response was defined as ≥50% reduction in VAS scores and ≥40% reduction in ODI scores at 3 months. The HALP score showed fair discriminative ability for both outcomes (VAS AUC = 0.717; ODI AUC = 0.731). The diagonal line represents the reference line of no discrimination (AUC = 0.5).

When HALP was evaluated as a single predictor, the ROC analysis demonstrated fair discrimination for predicting treatment response. For VAS response, the AUC was 0.717 (95% CI, 0.614–0.817) with an optimal cut-off of 39.8, yielding a sensitivity of 87.0% and a specificity of 58.1%. For ODI response, the AUC was 0.731 (95% CI, 0.627–0.831) with the same cut-off, providing a sensitivity of 87.3% and a specificity of 61.0% (Figure 3).

In exploratory analyses, we compared the discriminative performance of HALP with commonly used inflammatory ratios, including the neutrophil-to-lymphocyte ratio (NLR), platelet-to-lymphocyte ratio (PLR), and systemic immune–inflammation index (SII). HALP achieved markedly higher AUC values for both pain (VAS) and functional (ODI) responses (HALP = 0.717/0.731) compared with NLR (0.338/0.316), PLR (0.318/0.299), and SII (0.274/0.245), indicating superior discriminative performance (Table 5).

Consistent with these ROC-based results, applying HALP ≥39.8 as a simple classifier yielded higher responder rates than HALP < 39.8—VAS responders: 66/84 (78.6%) vs. 11/36 (30.6%), *p* < 0.001; ODI responders: 68/84 (81.0%) vs. 11/36 (30.6%), *p* < 0.001 (see Supplementary Table S2). In the multivariable logistic regression models that included HALP, along with age, sex, BMI, smoking status, paraspinal tenderness, and the corresponding baseline score, HALP remained a significant independent predictor of both outcomes (Table 3 and Table 4).

Sensitivity analyses within ±10% of the Youden-optimal HALP threshold (36–44) demonstrated stable performance. Sensitivity ranged from 0.73 to 0.90 and specificity from 0.41 to 0.66, while PPV and NPV remained between 0.75 and 0.81 and 0.56 and 0.69, respectively (Supplementary Table S3). These results indicate that the 39.8 threshold offers a clinically balanced trade-off between sensitivity and specificity.

**Figure 4:**
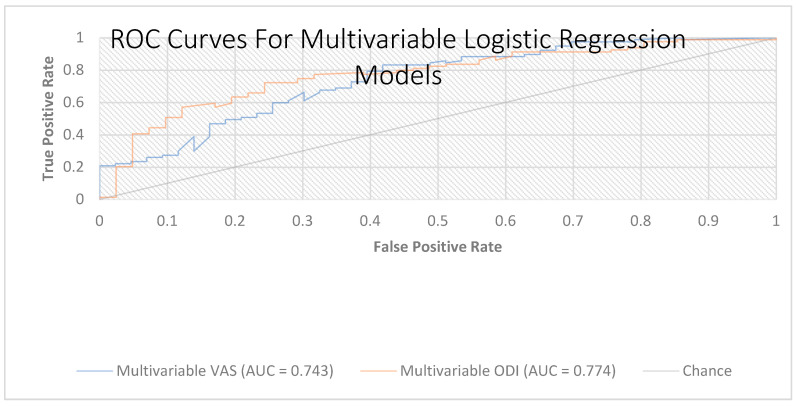
Receiver operating characteristic (ROC) curves for the multivariable logistic regression models (VAS and ODI). Receiver operating characteristic curves depicting the discrimination of two multivariable models, including HALP, age, sex, BMI, smoking status, paraspinal tenderness, and the corresponding baseline score (VAS or ODI). AUC = 0.744 for the VAS model and AUC = 0.774 for the ODI model. The diagonal line denotes the chance classifier; greater deviation above this line indicates better discrimination. Abbreviations: HALP, hemoglobin–albumin–lymphocyte–platelet score; VAS, Visual Analog Scale; ODI, Oswestry Disability Index; BMI, body mass index.

The ROC analysis of model-predicted probabilities showed apparent AUCs of 0.744 (VAS) and 0.774 (ODI), with optimism-corrected AUCs of 0.680 and 0.720, respectively (Figure 4).

Both models demonstrated good calibration (Hosmer–Lemeshow: VAS, *p* = 0.427; ODI, *p* = 0.936), with calibration slopes of 0.739 (VAS) and 0.784 (ODI) and Brier scores of 0.188 (VAS) and 0.179 (ODI), respectively, supporting the robustness of the predictive value of HALP (Table 6).

## 4. Discussion

In this cohort of patients undergoing medial branch RFA for degenerative facet–related chronic low back pain, higher pre-procedure HALP scores were independently associated with greater short-term improvements in both pain and disability. To our knowledge, this is the first study to evaluate HALP as a prognostic biomarker for response to lumbar medial branch RFA. Although HALP correlated with both outcomes, its discriminative performance was stronger for functional improvement (ODI) than for pain relief (VAS), suggesting that systemic immunonutritional status may be more closely linked to functional recovery. The Youden-optimal threshold of ~39.8 achieved high sensitivity but modest specificity, indicating potential clinical value primarily in identifying patients likely to respond.

Facet joints are paired synovial articulations that contribute to spinal stability, and facet-mediated pain accounts for nearly one-third of chronic spinal pain cases [11,12,13,14,15]. Degenerative osteoarthritis becomes increasingly prevalent with age, most commonly at the L4–L5 level, followed by the L3–L4 and L5–S1 levels. Clinically, pain often radiates toward the hip, groin, or thigh, and degenerative hypertrophy may mimic radiculopathy. Accordingly, accurate diagnosis requires integration of clinical examination, imaging, and comparative diagnostic injections [16,17,18,19,20]. In our study, the requirement of ≥50% relief after two diagnostic medial branch blocks, regarded as the reference standard, strengthened the validity of patient selection [19,20].

While some clinical factors, such as baseline VAS and paraspinal tenderness, showed weak trends, they were not significant predictors after adjustment, consistent with prior reports identifying paraspinal tenderness, long pain history, advanced imaging findings, or smoking status as relevant predictors [21,22]. These results underscore that, beyond technical accuracy, the selection of appropriate patients remains critical for sustained outcomes [23,24,25]. Baseline disability was modestly higher in patients with HALP < 39.8 compared with those with HALP ≥ 39.8, whereas other baseline demographics and scores were broadly comparable. Although such an imbalance could theoretically favor the high-HALP group, the independent association of HALP with both endpoints persisted after adjustment for baseline ODI, and stratified analyses using the ROC-derived cut-off (≥39.8) still showed substantially higher responder rates in the high-HALP group. Thus, the observed prognostic value of HALP is unlikely to be explained solely by baseline differences. Residual confounding by unmeasured psychosocial factors cannot be entirely excluded; however, defining responses as percentage reductions (VAS ≥ 50%, ODI ≥ 40%) helps limit regression-to-the-mean effects.

From a biological perspective, facet degeneration triggers local release of proinflammatory cytokines such as IL-1β, IL-6, IL-8, and TNF-α, which sensitize nociceptors and promote central sensitization [26,27]. HALP, as a composite index of hemoglobin, albumin, lymphocytes, and platelets, indirectly reflects systemic inflammatory balance, oxygen-carrying capacity, and nutritional/immune reserve. Higher HALP values may mitigate peripheral and central sensitization, thereby supporting improved outcomes [28]. Initially developed as a prognostic marker in oncology [8,9], HALP has subsequently demonstrated predictive value in other systemic conditions, including acute pancreatitis [10] and cardiovascular disease [29]. Our findings extend its application to interventional pain management, suggesting that HALP may serve as a secondary stratification tool after diagnostic blocks, potentially distinguishing true responders from false-positive block outcomes.

To further contextualize the prognostic relevance of HALP, exploratory comparisons were conducted with conventional inflammatory indices, including the neutrophil-to-lymphocyte ratio, platelet-to-lymphocyte ratio, and systemic immune–inflammation index. Consistent with prior pain-related studies, these markers demonstrated poor discriminative capacity, with AUCs ranging between 0.25 and 0.35 for both pain (VAS) and functional (ODI) outcomes. In contrast, HALP achieved markedly higher AUC values, indicating superior predictive ability. These findings suggest that HALP, which integrates both nutritional and immunologic components, may more comprehensively capture the multidimensional host status influencing radiofrequency outcomes than inflammation-based ratios alone.

The magnitude of discrimination observed in our cohort (AUC ≈ 0.71–0.73) closely aligns with previous reports of HALP in oncologic and cardiovascular prognostic studies, where AUCs generally range from 0.68 to 0.75 [8,9,10]. Conversely, the weak performance of NLR, PLR, and SII in our cohort is consistent with the limited and heterogeneous evidence in chronic pain and degenerative spine literature, where simple inflammation-based ratios often fail to predict long-term pain or functional outcomes [30,31]. For instance, Osunronbi et al. reported that preoperative NLR did not predict improvement in pain or disability at 12 months following lumbar fusion.

In contrast to Zhao et al. [32], who reported an AUC of 0.803 for NLR in predicting outcomes of intradiscal radiofrequency ablation for lumbar disk herniation, our cohort showed only minimal discriminative value (AUC ≈ 0.33). This discrepancy likely reflects the distinct inflammatory burden of the underlying pathology: while NLR performs well in acute or subacute discogenic inflammation, it appears less informative in chronic degenerative facet-related pain, where systemic immunonutritional status, as captured by HALP, may exert a more prominent prognostic role.

Moreover, NLR and PLR have been studied in postoperative and block-based settings primarily as markers of acute inflammatory response, but without clear prognostic value for functional recovery [32]. Taken together, these findings suggest that inflammation-based ratios may have limited translational relevance in chronic degenerative pain states. In contrast, composite indices such as HALP, which integrate both immunologic and nutritional components, may more effectively capture systemic resilience relevant to treatment outcomes.

Although HALP demonstrated statistical significance, the effect size per unit increase was modest (OR ≈ 1.06). Given HALP’s high sensitivity yet moderate specificity, HALP may serve better as a screening or triage biomarker rather than a definitive predictor. In clinical terms, a low HALP value may help identify patients less likely to benefit from RFA, whereas a high value alone does not guarantee response. Thus, HALP should complement, rather than replace, established selection criteria.

Clinically, incorporating HALP into patient selection could enhance counseling and resource allocation. Since HALP relies on routine laboratory parameters, it is inexpensive and easily integrated into practice. Patients with lower HALP scores may be counseled regarding a lower likelihood of success, while those with higher values may proceed with greater confidence.

This study has several strengths, including rigorous patient selection with double diagnostic blocks, standardized procedures, and performance by an experienced team. However, certain limitations must be acknowledged. Its retrospective, single-center design and relatively short three-month follow-up reduce external validity. Although the event-per-variable ratio (~11–13) met the conventional minimum threshold for logistic regression, the decline between apparent and optimism-corrected AUCs (0.74 → 0.68 for VAS; 0.77 → 0.72 for ODI) indicates mild model overfitting. Accordingly, these results should be interpreted as internally validated rather than externally generalizable, pending validation in larger, prospective cohorts. Psychosocial factors and detailed imaging parameters were not assessed. A modest baseline imbalance in ODI favored the ≥39.8 group; nevertheless, HALP remained independently associated with both outcomes after adjustment, and stratified analyses were concordant. HALP was measured only once, and its ROC-derived cut-off lacked external validation. This missing data resulted from an institutional change in laboratory policy rather than patient characteristics, as serum albumin was removed from the routine pre-procedure panel for cost-control reasons. Because of the retrospective design, this variability could not be standardized. All patients were outpatients, so significant systematic differences were unlikely. Nevertheless, prospective studies with uniform biochemical protocols are warranted to overcome this limitation. Although a 3-month follow-up was chosen to ensure uniform data completeness and minimize attrition bias, longer-term outcomes (6–12 months) would be valuable to confirm the durability of HALP’s predictive performance over time. Larger, prospective multicenter studies with long-term outcomes are warranted.

## 5. Conclusions

In conclusion, these findings support the testable premise that incorporating the simple, inexpensive, and widely available HALP score into post-block triage may enrich for responders particularly for functional (ODI) improvement to lumbar medial branch RFA; however, before routine clinical adoption, prospective multicenter trials should verify cut-offs, quantify net clinical benefit, and assess whether correcting low HALP states enhances outcomes.

## Figures and Tables

**Table 1:** Baseline demographic and clinical characteristics of responders and non-responders according to the VAS response criterion.

Variable/ +	Responders (*n* = 77)	Non-Responders (*n* = 43)	*p*-Value
Age	68.81 ± 5.90	68.77 ± 7.20	0.773
Baseline VAS	8.60 ± 0.91	8.30 ± 0.86	0.130
Baseline ODI	22.14 ± 4.99	20.95 ± 3.55	0.510
BMI	30.37 ± 2.62	30.72 ± 3.15	0.368
HALP score	54.61 ± 14.96	43.35 ± 14.68	<0.001
Paraspinal tenderness	66 (85.7%)	31 (72.1%)	0.115
Smoking	38 (49.4%)	18 (41.9%)	0.550
Sex (Female)	42 (54.5%)	26 (60.5%)	0.663

Values are expressed as mean ± standard deviation for continuous variables and number (percentage) for categorical variables. Group comparisons were performed using the Mann–Whitney U test for continuous variables and the chi-square/Fisher test for categorical variables. A *p*-value < 0.05 was considered statistically significant. VAS, Visual Analog Scale; ODI, Oswestry Disability Index; BMI, Body Mass Index; HALP, Hemoglobin–Albumin–Lymphocyte–Platelet.

**Table 2:** Baseline demographic and clinical characteristics of responders and non-responders according to the ODI response criterion.

Variable	Responders (*n* = 79)	Non-Responders (*n* = 41)	*p*-Value
Age	68.73 ± 5.84	68.90 ± 7.34	0.898
Baseline VAS	8.61 ± 0.90	8.27 ± 0.87	0.067
Baseline ODI	22.06 ± 4.95	21.05 ± 3.60	0.660
BMI	30.34 ± 2.64	30.78 ± 3.15	0.318
HALP score	54.65 ± 14.83	42.72 ± 14.62	<0.001
Paraspinal tenderness	68 (86.1%)	29 (70.7%)	0.075
Smoking	39 (49.4%)	17 (41.5%)	0.529
Sex (Female)	44 (55.7%)	24 (58.5%)	0.918

Values are expressed as mean ± standard deviation for continuous variables and number (percentage) for categorical variables. Group comparisons were performed using the Mann–Whitney U test for continuous variables and the chi-square/Fisher test for categorical variables. A *p*-value < 0.05 was considered statistically significant. Abbreviations: VAS, Visual Analog Scale; ODI, Oswestry Disability Index; BMI, Body Mass Index; HALP, Hemoglobin–Albumin–Lymphocyte–Platelet.

**Table 3:** Multivariable logistic regression analysis according to the ODI response criterion.

Variable	OR	95% CI	*p*-Value
HALP score	1.06	1.02–1.09	<0.001
Age	1.00	0.94–1.07	0.952
Sex	0.97	0.42–2.26	0.948
BMI	0.98	0.85–1.13	0.779
Smoking	1.22	0.53–2.82	0.645
Paraspinal tenderness	2.13	0.78–5.88	0.142

Multivariable logistic regression was performed to identify independent predictors of treatment response, defined as a ≥40% improvement in the Oswestry Disability Index (ODI). The model included the HALP score, age, sex, body mass index (BMI), smoking status, and presence of paraspinal tenderness as covariates. Odds ratios (OR) are presented with corresponding 95% confidence intervals (CI) and *p*-values. A *p*-value <0.05 was considered statistically significant. ODI, Oswestry Disability Index; HALP, Hemoglobin–Albumin–Lymphocyte–Platelet; BMI, Body Mass Index; OR, Odds Ratio; CI, Confidence Interval.

**Table 4:** Multivariable logistic regression analysis according to the VAS response criterion.

Variable	OR	95% CI	*p*-Value
HALP score	1.05	1.02–1.08	0.001
Age	1.01	0.95–1.08	0.793
Sex	1.14	0.50–2.61	0.761
BMI	0.99	0.86–1.14	0.874
Smoking	1.21	0.53–2.75	0.647
Paraspinal tenderness	1.96	0.72–5.34	0.190

Multivariable logistic regression was conducted to identify independent predictors of treatment response, defined as a ≥ 0% reduction in the Visual Analog Scale (VAS) score. The model included the HALP score, age, sex, body mass index (BMI), smoking status, and presence of paraspinal tenderness as covariates. Odds ratios (OR) are presented with corresponding 95% confidence intervals (CI) and *p*-values. A *p*-value <0.05 was considered statistically significant. VAS, Visual Analog Scale; HALP, Hemoglobin–Albumin–Lymphocyte–Platelet; BMI, Body Mass Index; OR, Odds Ratio; CI, Confidence Interval.

**Table 5:** Comparative ROC performance of HALP versus conventional inflammatory indices for predicting 3-month treatment response.

Marker	AUC (VAS)	95% CI (VAS)	AUC (ODI)	95% CI (ODI)	Sensitivity	Specificity
HALP score	0.717	0.61–0.82	0.731	0.63–0.83	0.87	0.61
NLR (Neutrophil-to-Lymphocyte Ratio)	0.338	0.26–0.41	0.316	0.25–0.39	—	—
PLR (Platelet-to-Lymphocyte Ratio)	0.318	0.24–0.38	0.299	0.23–0.397	—	—
SII (Systemic Immune–Inflammation Index)	0.274	0.20–0.33	0.245	0.18–0.31	—	—

HALP showed higher AUCs than NLR, PLR, and SII for both endpoints. For inverse markers (where a higher value indicates a worse outcome), direction was aligned for comparability. AUC—Area Under the Receiver-Operating Characteristic curve; VAS—Visual Analogue Scale; ODI—Oswestry Disability Index; HALP—Hemoglobin-Albumin-Lymphocyte-Platelet; NLR—Neutrophil-to-Lymphocyte Ratio; PLR—Platelet-to-Lymphocyte Ratio; SII—Systemic Immune–Inflammation Index.

**Table 6:** Discriminative performance and calibration of HALP alone versus multivariable logistic regression models for predicting treatment response.

Model	AUC	AUC (Optimism-Corrected)	Hosmer–Lemeshow *p*	Calibration Slope	Brier Score
HALP only (VAS)	0.717	–	–	–	–
HALP only (ODI)	0.731	–	–	–	–
Multivariable (VAS)	0.744	0.680	0.427	0.739	0.188
Multivariable (ODI)	0.774	0.720	0.936	0.784	0.179

Values represent apparent and optimism-corrected AUCs (bootstrap 500 iterations), Hosmer–Lemeshow goodness-of-fit *p*-values, and calibration slopes. HALP = hemoglobin, albumin, lymphocyte, platelet score; ODI = Oswestry Disability Index; VAS = Visual Analog Scale.

## Data Availability

The data that support the findings of this study are available from the corresponding author upon reasonable request. The data are not publicly available due to institutional policy and privacy restrictions.

## Supplementary Materials

The following supporting information can be downloaded at: https://www.mdpi.com/article/10.3390/diagnostics15212758/s1, Table S1. Baseline characteristics by HALP cut-off (<39.8 vs. ≥39.8); Table S2. Clinical outcomes by HALP group (<39.8 vs ≥39.8); Table S3. Robustness of HALP cut-off (±10% around the Youden threshold) for predicting functional response (ODI ≥ 40%).

## Abbreviations

AUC: Area Under the Curve, CI: Confidence Interval, HALP: Hemoglobin, Albumin, Lymphocyte, Platelet, IQR: Interquartile Range, MBB: Medial Branch Block, ODI: Oswestry Disability Index, OR: Odds Ratio, RF: Radiofrequency, ROC: Receiver Operating Characteristic, SD: Standard Deviation, VAS: Visual Analog Scale, VIF: Variance Inflation Factor
