# Supplementary material for: Association of the Hemoglobin–Albumin–Lymphocyte–Platelet (HALP) Score with 3-Month Outcomes After Lumbar Medial Branch Radiofrequency Ablation: A Retrospective Cohort Study
Source: Diagnostics (Basel). 2025 Oct 31;15(21):2758. doi: 10.3390/diagnostics15212758 (PMC12607953; doi:10.3390/diagnostics15212758)
Supplement: Supplementary file 1 [file diagnostics-15-02758-s001.zip › TABLE S1.pdf]

Supplementary Table S1. Baseline characteristics by HALP group (< 39.8 vs ≥ 39.8)

Variable	<39.8 (n=36)	≥39.8 (n=84)	Test	p-value
**Age**	69.08 ± 6.73	68.67 ± 6.24	Mann–Whitney U	0.658
**BMI**	31.03 ± 2.79	30.27 ± 2.81	Mann–Whitney U	0.077
**VAS baseline**	8.58 ± 0.87	8.45 ± 0.91	Mann–Whitney U	0.344
**ODI baseline**	23.03 ± 4.53	21.15 ± 4.47	Mann–Whitney U	0.026
**Male, n (%)**	14/36 (38.9%)	38/84 (45.2%)	Chi-square	0.520
**Smoker, n (%)**	14/36 (38.9%)	42/84 (50.0%)	Chi-square	0.264
**Paraspinal tenderness present, n (%)**	28/36 (77.8%)	69/84 (82.1%)	Chi-square	0.578

Notes: Continuous variables are presented as mean ± SD and compared using the Mann–Whitney U test; categorical variables are presented as n/N (%) and compared using Pearson's χ^2 test (Fisher's exact test when expected counts were < 5). Two-sided p-values are reported. HALP groups were defined by the Youden-optimal cut-off of 39.8 (≥ 39.8 considered favorable). "Male" indicates sex = male; "Smoker" indicates cigarette use = yes; "Paraspinal tenderness present" indicates paraspinal tenderness = yes.

Abbreviations: HALP, hemoglobin–albumin–lymphocyte–platelet score; BMI, body mass index; VAS, Visual Analog Scale; ODI, Oswestry Disability Index.
