# Supplementary material for: Association of the Hemoglobin–Albumin–Lymphocyte–Platelet (HALP) Score with 3-Month Outcomes After Lumbar Medial Branch Radiofrequency Ablation: A Retrospective Cohort Study
Source: Diagnostics (Basel). 2025 Oct 31;15(21):2758. doi: 10.3390/diagnostics15212758 (PMC12607953; doi:10.3390/diagnostics15212758)
Supplement: Supplementary file 1 [file diagnostics-15-02758-s001.zip › TABLE S2.pdf]

Supplementary Table S2. Clinical outcomes by HALP group (< 39.8 vs ≥ 39.8)

Outcome	<39.8 responders	≥39.8 responders	Test	p-value	Odds ratio (≥39.8 vs <39.8)
**VAS ≥50%**	11/36 (30.6%)	66/84 (78.6%)	Chi-square	<0.001	8.33 (3.46–20.09)
**ODI ≥40%**	11/36 (30.6%)	68/84 (81.0%)	Chi-square	<0.001	9.66 (3.95–23.62)

Notes: Responder rates at 3 months are reported as n/N (%) for VAS ≥ 50% pain reduction and ODI ≥ 40% disability reduction. Between-group differences were assessed using Pearson's χ^2 test (Fisher's exact test when expected counts were < 5). Odds ratios (95% CI) compare HALP ≥ 39.8 to HALP < 39.8; all p-values are two-sided. The 39.8 threshold was derived as the Youden-optimal cut-off from the HALP-only ROC analysis.
