# Supplementary material for: Association of the Hemoglobin–Albumin–Lymphocyte–Platelet (HALP) Score with 3-Month Outcomes After Lumbar Medial Branch Radiofrequency Ablation: A Retrospective Cohort Study
Source: Diagnostics (Basel). 2025 Oct 31;15(21):2758. doi: 10.3390/diagnostics15212758 (PMC12607953; doi:10.3390/diagnostics15212758)
Supplement: Supplementary file 1 [file diagnostics-15-02758-s001.zip › TABLE S3.pdf]

Supplementary Table S3. Robustness of HALP cut-off (±10% around the Youden threshold) for predicting functional response (ODI ≥ 40%).

HALP Cut-off	Sensitivity	Specificity	PPV	NPV
**36.0**	0.90	0.41	0.75	0.68
**39.8 (Youden-optimal)**	0.86	0.61	0.81	0.69
**44.0**	0.73	0.66	0.81	0.56

** Sensitivity analyses within ±10% of the Youden-optimal threshold (36–44) demonstrated stable predictive performance. Sensitivity ranged from 0.73 to 0.90 and specificity from 0.41 to 0.66, while PPV and NPV remained between 0.75–0.81 and 0.56–0.69, respectively. HALP – Hemoglobin-Albumin-Lymphocyte-Platelet; ODI – Oswestry Disability Index; PPV – Positive Predictive Value; NPV – Negative Predictive Value.*
